# Practical approaches to Bayesian sample size determination in non-inferiority trials with binary outcomes

**DOI:** 10.1002/sim.9661

**Published:** 2023-01-20

**Authors:** Rebecca M. Turner, Michelle N. Clements, Matteo Quartagno, Victoria Cornelius, Suzie Cro, Deborah Ford, Conor D. Tweed, A. Sarah Walker, Ian R. White

**Affiliations:** 1 MRC Clinical Trials Unit, University College London, London, UK; 2 Imperial Clinical Trials Unit, School of Public Health, Imperial College London, London, UK

**Keywords:** Bayesian methods, non-inferiority trials, sample size, trial design

## Abstract

Bayesian analysis of a non-inferiority trial is advantageous in allowing direct probability statements to be made about the relative treatment difference rather than relying on an arbitrary and often poorly justified non-inferiority margin. When the primary analysis will be Bayesian, a Bayesian approach to sample size determination will often be appropriate for consistency with the analysis. We demonstrate three Bayesian approaches to choosing sample size for non-inferiority trials with binary outcomes and review their advantages and disadvantages. First, we present a predictive power approach for determining sample size using the probability that the trial will produce a convincing result in the final analysis. Next, we determine sample size by considering the expected posterior probability of non-inferiority in the trial. Finally, we demonstrate a precision-based approach. We apply these methods to a non-inferiority trial in antiretroviral therapy for treatment of HIV-infected children. A predictive power approach would be most accessible in practical settings, because it is analogous to the standard frequentist approach. Sample sizes are larger than with frequentist calculations unless an informative analysis prior is specified, because appropriate allowance is made for uncertainty in the assumed design parameters, ignored in frequentist calculations. An expected posterior probability approach will lead to a smaller sample size and is appropriate when the focus is on estimating posterior probability rather than on testing. A precision-based approach would be useful when sample size is restricted by limits on recruitment or costs, but it would be difficult to decide on sample size using this approach alone.

## 1 Introduction

Non-inferiority trials evaluate treatments which are not expected to be more effective than the standard treatment, but which may provide other benefits, for example, with respect to adverse effects, costs, or convenience. Non-inferiority designs are increasingly common in clinical trials,^1^ because use of placebo or control treatments in a trial is unethical when an effective standard treatment has already been established. A non-inferiority trial is designed to test whether the efficacy of the new treatment is not unacceptably lower than that of the standard treatment and to provide evidence of superiority on other outcomes such as safety or acceptability to patients. At the design stage, trial investigators pre-specify their chosen non-inferiority margin for loss of efficacy from using the new treatment, representing the null hypothesis to be tested in the trial.

Bayesian approaches for analysis of non-inferiority trials have been proposed by several authors.^2–5^ An important advantage of the Bayesian framework is that it allows a probabilistic interpretation of all trial results; in non-inferiority trials we can therefore make direct probability statements about the non-inferiority of the new treatment. In the common situation, where achieving consensus over the choice of margin is difficult and opinions on the most suitable value differ among investigators, probability statements could be reported for several non-inferiority margins rather than one fixed margin. A second advantage is that a Bayesian approach allows us to explore the impact of varying opinions over likely values of the treatment effect on the analysis, through incorporating informative priors. Incorporating relevant external evidence is particularly valuable in settings such as rare diseases, rare disease subtypes, or pediatric populations, where recruitment of sufficient numbers of patients to achieve the large sample sizes often required for a non-inferiority trial may be difficult.^6^

When designing a trial, the sample size calculations should be based on the planned primary analysis. If a Bayesian analysis is planned, either with informative or non-informative priors, it would be preferable for consistency with the analysis to choose the sample size under a Bayesian framework. A Bayesian approach to sample size determination makes appropriate allowance for the uncertainty over design parameters such as treatment effect and the non-inferiority margin, rather than assuming that these values are fixed and known. If an informative prior will be used in the analysis, this should ideally be taken into account in the trial design by using a Bayesian sample size calculation. It is crucial that the sample size calculation can be explained and justified to clinicians and funders as well as being appropriate.

Bayesian approaches to sample size determination have been widely discussed and include methods analogous to the frequentist power-based approach, approaches based on examining precision of the effect of interest and decision-theoretic approaches.^7–12^ Most illustrative examples in the Bayesian design literature relate to superiority trials; here, we focus on how to determine sample size using Bayesian methods in the special case of non-inferiority designs and explore three available approaches. We review the advantages and disadvantages of using these in practice, and make recommendations. We assume throughout that a Bayesian rather than frequentist analysis is planned. We emphasize the importance of taking into account prior uncertainty in the true treatment effect rather than regarding this as known as is usual under a frequentist approach, and also explore the option of providing an informative prior for the non-inferiority margin, which could be based on expert elicitation.^13^ For convenience we assume a binary outcome, but the concepts (if not the computational details) apply to other outcome types.

First, we choose sample size on the basis of the probability that the trial will produce a specified level of evidence supporting non-inferiority in a Bayesian non-inferiority analysis, analogous to frequentist power. In a second approach, we determine sample size by considering the expected posterior probability of non-inferiority in the trial. Next, we take a precision-based approach and choose sample size by examining the relationship between sample size and the resulting precision of the posterior distribution for the treatment effect. The characteristics of the sample size determination approaches considered are summarized in Table 1. We apply these methods to an example non-inferiority trial design and make comparison with a standard frequentist approach to calculating sample size.

## 2 Methods

Suppose that the trial design includes the intention that a Bayesian analysis will be carried out, with the following analysis prior distributions specified for the event probabilities *π*_1_ and $\pi_{0}:\pi_{1}\sim \text{Beta }(a_{1}^{A},b_{1}^{A})$ and $\pi_{0}\sim \text{Beta }\left(a_{0}^{A},b_{0}^{A}\right)$. The prior distributions that will be used in the planned Bayesian analysis could be vague or informative^9^ and will be referred to as the “analysis prior.” Separately, we can use a “design prior” to represent uncertainty at the design stage: $\pi_{1}\sim \text{Beta }\left(a_{1}^{D},b_{1}^{D}\right)$ and $\pi_{0}\sim \text{Beta }\left(a_{0}^{D},b_{0}^{D}\right)$. The design prior and analysis prior need not be the same; for example, researchers might be willing to make use of external information about the treatment effect when designing the trial but not when performing the analysis. The posterior distributions for *π*_1_ and *π*_0_ are $\text{Beta }(a_{1}^{A}+r_{1},b_{1}^{A}+n-r_{1})$ and $\text{Beta }\left(a_{0}^{A}+r_{0},b_{0}^{A}+n-r_{0}\right)$, where *r*_1_, *r*_0_ represent the observed numbers of events in the two trial arms and each treatment arm has *n* patients.

### 2.1 Bayesian power approach

The Bayesian approach that is closest in perspective to the standard frequentist approach for determining sample size is based on examining the probability that the trial will produce a specified level of evidence supporting non-inferiority in the planned Bayesian analysis. Spiegelhalter et al^8^ have recommended considering the unconditional probability obtained by averaging the power curve with respect to prior distributions for unknown parameters. In a non-inferiority trial, we would calculate the predictive probability of obtaining a “significant” Bayesian result (defined below) when comparing the null hypothesis *δ* ≥ *δ*^∗^ against the alternative hypothesis *δ* < *δ*^∗^, where *δ* is the treatment effect and *δ*^∗^ is the (positive) non-inferiority margin, assuming the trial outcome is unfavorable. A significant Bayesian result is where the posterior probability of non-inferiority exceeds a pre-specified threshold: P(*δ* < *δ*^∗^|data) > 1 − *α*/2, where *α* is the chosen two-sided significance level.

We will focus on the probability of obtaining a significant Bayesian result and of this being the correct decision (ie, *π*_1_ and *π*_0_ represent non-inferiority) rather than on the unconditional probability of a significant result, since the latter could potentially include rejections of the null hypothesis under inferiority that represent a type I error.^14^ In practice, the difference between the two probabilities is likely to be negligible in most situations, assuming that prior opinion at the design stage is supportive of non-inferiority, since the prior probability of superiority is likely to be small.^8^

We will refer to the probability that a trial will produce a significant Bayesian result and that this decision is correct as the predictive power. The sample size can be chosen with the aim of achieving a desired predictive power while allowing for uncertainty in the treatment effect, rather than achieving a desired power conditional on an assumed fixed treatment effect, as in the standard frequentist calculations.

For trials with binary outcomes, the predictive probability of a significant Bayesian result is averaged over design prior distributions for the unknown event rates *π*_1_ and *π*_0_ in both treatment arms, chosen to reflect uncertainty at the design stage.

We assume that the treatment effect and non-inferiority margin are defined on the absolute (risk difference) scale. In a trial including *n* patients in each treatment arm, with true event probabilities *π*_1_ and *π*_0_, the probability of rejecting the null hypothesis *δ* ≥ *δ*^∗^ in a Bayesian analysis and of this being the correct decision (jointly denoted as rejection event *R*) is:

(1) $$\begin{array}{c} \text{P}(R|\pi_{1},\pi_{0})=E_{r_{1},r_{0}}{ℐ}_{S}(r_{1},r_{0},n,\delta^{*}){ℐ}_{NI}(\pi_{1},\pi_{0}),\\ {ℐ}_{S}(r_{1},r_{0},n,\delta^{*})=\{\begin{array}{cc} 1 & \;\text{if}\;\frac{\delta^{*}-(E(\pi_{1}|r_{1})-E(\pi_{0}|r_{0}))}{\sqrt{Var(\pi_{1}|r_{1})+Var(\pi_{0}|r_{0})}}>Z_{\alpha /2}\\ 0 & \;\text{otherwise},\; \end{array} \end{array}$$

where ℐ_*NI*_ is an indicator function taking the value 1 if *π*_1_ − *π*_0_ < *δ*^∗^ and 0 otherwise. The expression ℐ_*S*_ (*r*_1_, *r*_0_, *n, δ*^∗^) indicates whether the null hypothesis is rejected in a Bayesian analysis incorporating the chosen analysis priors, while ℐ_*NI*_ indicates whether a significant result is correct. A normal approximation has been used for the posterior distribution of the treatment effect *δ* = *π*_1_ − *π*_0_, with mean and variance calculated from the posterior means and variances of *π*_1_ and *π*_0_. For a given sample size *n*, the expectation is taken over data values *r*_1_~Bin (*n, π*_1_), *r*_0_~Bin (*n, π*_0_) sampled from the design prior distributions. The expectation in (1) is the joint unconditional probability that the trial will produce a significant Bayesian result and that this decision is correct.^8^

Under this approach, we can choose between assuming a fixed non-inferiority margin *δ*^∗^, as is usual under the frequentist approach to sample size calculation, or specifying a prior to represent clinical uncertainty (variability of opinion among experts) about *δ*^∗^, which could be informed by elicitation of expert clinical opinion. Aupiais et al elicited experts’ views on acceptable differences between trial arm event rates and used these to construct prior distributions for the non-inferiority margin.^13^ We note that the prior should represent variability of opinion among experts rather than uncertainty within a single expert. In expression (1), the rejection event *R* then represents rejection of the null hypothesis *δ* ≥ *δ*^∗^ conditional on *δ*^∗^ and the expectation represents the expected proportion of experts who are convinced by the result of the Bayesian analysis.

### 2.2 Expected posterior probability approach

Under the previous approach, we choose sample size on the basis of the predictive probability of obtaining a significant result in the analysis. As an alternative approach, some authors have suggested basing sample size on the expected posterior probability for superiority of one treatment over the other.^15,16^ Here, we consider using an equivalent approach for non-inferiority trials.

Let *Y*_*n*_ be an estimator of the treatment effect *δ* = *π*_1_ − *π*_0_, based on *n* observations, and let *p*_*n*_ denote the predictive density of *Y*_*n*_, based on the design prior distributions for *π*_1_ and *π*_0_. To choose a sample size, we now examine the expected value of the posterior probability of non-inferiority for a fixed non-inferiority margin *δ*^∗^, with respect to the predictive density of the data:

(2) $$e_{n}={\text{E}}_{p_{n}}\left[P\left(\delta <\delta^{*}|\text{data}\;\right)\right].$$

We then choose the minimum sample size *n* to provide an acceptable value for the expected probability of non-inferiority. This approach allows us to control the expectation of the posterior probability distribution for non-inferiority, whereas the Bayesian power approach additionally controls the variability of the distribution. The sample sizes chosen under an expected posterior probability approach will be smaller than those chosen under a Bayesian power approach.

As in the Bayesian power approach, we need to take into account the analysis prior distribution planned to be used for the treatment effect in the planned Bayesian analysis, which could be vague or informative. We can also choose between assuming a fixed non-inferiority margin *δ*^∗^ or specifying a prior to represent clinical uncertainty about *δ*^∗^, which could be informed by elicitation of expert opinion.

### 2.3 Precision-based approach

An alternative Bayesian approach to determining sample size is to consider the implications of various sample sizes for the precision of the posterior distribution of the treatment effect *δ*. Previously suggested approaches include choosing sample sizes that achieve suitably narrow 95% credible intervals for the parameter of interest, that achieve high coverage for a fixed credible interval width, or that achieve a desirable combination of interval width and coverage.^11^ Focusing on achieving suitably narrow 95% credible intervals for the treatment effect would be the most accessible of these approaches in an applied trial setting. We note that a precision-based approach to determining sample size would be the same regardless of whether the trial is designed to assess non-inferiority or superiority of a new treatment in comparison with a standard treatment. The precision of the posterior distribution for *δ* is dependent on the sample size and the prior distributions to be used in the Bayesian analysis; design prior distributions are not incorporated in this approach.

When the sample size of a planned non-inferiority trial is restricted for cost reasons or by limits on expected recruitment, a precision-based approach could be used to demonstrate how much precision a fixed sample size would provide for the treatment effect estimate. If sample size can be chosen without restrictions, we can plot the 95% credible interval width for the treatment effect against a range of possible sample sizes and choose sample size according to the desired interval width. The process for justifying choice of a particular interval width is less clear if sample size is unrestricted, so it may be preferable to use a precision-based approach alongside a Bayesian power approach.

When assuming Beta analysis priors for the event probabilities in each trial arm, as in the previous approaches, conjugacy for the binomial distribution allows the credible intervals to be calculated analytically. The risk difference can then be assumed approximately normal, with mean and variance calculated from the Beta posterior distributions for *π*_1_ and *π*_0_:

(3) $$\delta |r_{1},r_{0}\sim N\left(E\left(\pi_{1}|r_{1}\right)-E\left(\pi_{0}|r_{0}\right),Var\left(\pi_{1}|r_{1}\right)+Var\left(\pi_{0}|r_{0}\right)\right).$$

This makes it straightforward to plot the relationship between 95% credible interval width and sample size, without the need for simulations. The analysis priors chosen for the event probabilities could correspond to a vague or informative prior for the treatment effect. Under the assumption of normality for the posterior distribution, the 100(1 − *α*)% credible interval width for the risk difference *δ* is:

(4) $$2Z_{\alpha /2}\sqrt{\frac{\left(a_{1}^{A}+np_{1}\right)\left(b_{1}^{A}+n\left(1-p_{1}\right)\right)}{{\left(a_{1}^{A}+b_{1}^{A}+n\right)}^{2}\left(a_{1}^{A}+b_{1}^{A}+n+1\right)}+\frac{\left(a_{0}^{A}+np_{0}\right)\left(b_{0}^{A}+n\left(1-p_{0}\right)\right)}{{\left(a_{0}^{A}+b_{0}^{A}+n\right)}^{2}\left(a_{0}^{A}+b_{0}^{A}+n+1\right)}},$$

where *p*_1_ and *p*_0_ are the assumed observed failure proportions in both arms (set equal to the prior means for *π*_1_ and *π*_0_). By plotting 95% credible interval width against sample size *n*, we can determine which sample size would provide a desirable level of precision and how much precision would be gained by increasing sample size.

As an additional representation of precision of the information provided by a particular sample size, we consider implementing ACceptability Curve Estimation using Probability above Threshold (ACCEPT). ACCEPT involves examining the cumulative probability of the true risk difference being greater than a given non-inferiority threshold, for a range of threshold values.^17^ Here, we plot ACCEPT curves for non-inferiority threshold values between 5% and 15%, assuming observed failure proportions *p*_1_ and *p*_0_ as above. We again assume Beta analysis priors for the event probabilities and assume the risk difference *δ* to be approximately normal, with mean and variance calculated from Beta posterior distributions for risk. The posterior probability of *δ* being greater than a given non-inferiority threshold *δ*^∗^ is:

(5) $$\begin{array}{c} \text{P}\left(\delta >\delta^{*}|\text{data}\right)=1-\text{Φ}\left[\frac{\delta^{*}-\mu_{\delta }}{\sigma_{\delta }}\right],\\ \mu_{\delta }=\frac{a_{1}^{A}+np_{1}}{a_{1}^{A}+b_{1}^{A}+n}-\frac{a_{0}^{A}+np_{0}}{a_{0}^{A}+b_{0}^{A}+n},\\ \sigma_{\delta }=\sqrt{\frac{\left(a_{1}^{A}+np_{1}\right)\left(b_{1}^{A}+n\left(1-p_{1}\right)\right)}{{\left(a_{1}^{A}+b_{1}^{A}+n\right)}^{2}\left(a_{1}^{A}+b_{1}^{A}+n+1\right)}+\frac{\left(a_{0}^{A}+np_{0}\right)\left(b_{0}^{A}+n\left(1-p_{0}\right)\right)}{{\left(a_{0}^{A}+b_{0}^{A}+n\right)}^{2}\left(a_{0}^{A}+b_{0}^{A}+n+1\right)}}, \end{array}$$

For a fixed sample size *n*, the ACCEPT curve is a plot of the posterior probability (5) against a range of non-inferiority thresholds *δ*^∗^. We plot a set of ACCEPT curves for different possible sample sizes and compare these, to consider whether there is a point beyond which little interpretability is gained by increasing sample size. We consider sample sizes that would provide 90% power under a frequentist sample size calculation (using (6) below) assuming each of five plausible non-inferiority margins *δ*^∗^ = {5%, 7.5%, 10%, 12.5%, 15%} respectively, that is n = {1241,551,310,199,138}.

### 2.4 Frequentist approach

For comparison, we use a standard frequentist approach to calculating the sample size required for a non-inferiority trial with a binary outcome. Let *π*_*e*1_ and *π*_*e*0_ be the expected event rates assumed in the sample size calculation. Then the number *n* of patients required in each treatment arm is:

(6) $$\begin{array}{c} n={\left(Z_{\alpha /2}+Z_{\beta }\right)}^{2}\left[1/f\left(\pi_{e1},\pi_{e0},\delta^{*}\right)\right],\\ f\left(\pi_{e1},\pi_{e0},\delta^{*}\right)=\frac{{\left(\pi_{e1}-\pi_{e0}-\delta^{*}\right)}^{2}}{\pi_{e1}\left(1-\pi_{e1}\right)+\pi_{e0}\left(1-\pi_{e0}\right)}. \end{array}$$

### 2.5 Implementation

The Bayesian power approach is implemented by sampling parameters *π*_1_ and *π*_0_ from their assumed design prior distributions $\pi_{1}\sim \text{Beta }\left(a_{1}^{D},b_{1}^{D}\right),\pi_{0}\sim \text{Beta }\left(a_{0}^{D},b_{0}^{D}\right)$, and data values *r*_1_~Bin (*n, π*_1_), *r*_0_~Bin (*n, π*_0_), using Monte Carlo simulation. We calculate the expectation of indicator functions representing rejection of the null hypothesis *δ* ≥ δ* in the planned Bayesian analysis incorporating the chosen analysis priors $\pi_{1}\sim \text{Beta }\left(a_{1}^{A},b_{1}^{A}\right)$ and $\pi_{0}\sim \text{Beta }\left(a_{0}^{A},b_{0}^{A}\right)$, and this being the correct decision (ie, *π*_1_ and *π*_0_ represent non-inferiority). This provides the unconditional joint probability that the trial will produce a significant Bayesian result and that this decision is correct.

The expected posterior probability approach is implemented by sampling data values *r*_1_, *r*_0_ as above. This provides a distribution for the posterior probability of non-inferiority, incorporating the chosen analysis priors. The expected value of this distribution is the expected posterior probability of non-inferiority.

For the precision-based approach, the 95% credible interval width for the risk difference (provided in (4)) is plotted against sample size, to examine which sample size would provide a desirable level of precision. In addition, we examine ACCEPT curves for different possible sample sizes, as an extra representation of the precision provided by a particular sample size. The frequentist calculations are carried out analytically using formula (6).

Code for implementing all approaches is provided in supplementary material.

## 3 Application to an Example

We revisit the sample size calculation for the ODYSSEY trial, which is a non-inferiority trial comparing dolutegravir-based antiretroviral therapy to standard of care for treatment of HIV-infected children; the trial was ongoing at the time of writing^18^ but has since been completed.^19^ We explore how the sample size could be chosen if a Bayesian analysis were planned for the trial. The primary outcome of the trial is virological or clinical failure by 96 weeks and a failure proportion of 18% was anticipated in both treatment arms. Treatment effect will be measured on the risk difference scale and a non-inferiority margin of 10% was chosen, informed by previous studies.^18^ Under a standard frequentist approach, 310 patients per arm will provide 90% power for excluding a difference of more than 10% between equivalent treatment arms, using a two-sided 95% confidence interval. A sample size of 700 was chosen for the ODYSSEY trial to allow for 10% loss to follow-up.^18^ Results are summarized in Table 2.

We assume initially that wide analysis prior distributions will be used in the planned Bayesian analysis, and assume a fixed non-inferiority margin of 10%. Vague priors are usually preferred for the treatment effect in analysis, in order that the posterior distribution is influenced primarily by observed data. As analysis priors, we specify flat Beta (1,1) distributions for both failure proportions. These distributions represent lack of prior information.

Under a Bayesian power approach, we examine the predictive probability of obtaining a significant Bayesian result, while acknowledging uncertainty in the failure proportions in both treatment arms. As in the frequentist approach, we suppose that the expected failure proportion is 18% in each arm, but whereas in a frequentist approach these values are assumed fixed and known (although unknown in practice), in a Bayesian approach we now assume design priors with SDs of 2% to represent some uncertainty around these values. Choice of SD could be informed by previous related studies (eg, pediatric trials where similar standard of care has been used or adult trials comparing the same treatments as ODYSSEY) or by elicitation from clinical experts. By matching moments, we choose a Beta(66,302) distribution to represent prior information for the failure proportions *π*_1_ and *π*_0_ at the design stage; this has a mean of 18% and 95% range of 14% to 22%. These distributions represent strong prior information and the prior for each failure proportion has an effective sample size equivalent to 368 individuals.^20^

With a sample size of 310 patients per group, from the frequentist sample size calculation with 90% power, the predictive power of concluding non-inferiority for dolutegravir compared to standard of care is 83% using Monte Carlo simulation. The predictive power allowing for uncertainty in expected failure proportions will usually be lower than the power calculated assuming fixed values for failure proportions used in a frequentist approach. Since the power based on fixed failure proportions is 90%, the power corresponding to each of a range of failure proportions could be substantially lower than 90% but up to only 10% higher (depending on the prior distributions chosen for the failure proportions). The predictive power allowing for uncertainty in expected failure proportions is the mean power value and is therefore usually lower than frequentist power. A sample size of 440 patients per group would be needed to provide 90% predictive power for concluding non-inferiority. To obtain Bayesian power that matches frequentist power, we would need to specify design priors with the same mean and a very small variance, for example Beta(6600, 30 200) priors. When assuming these together with flat Beta (1,1) analysis priors, a sample size of 310 per group provides predictive power of 90%, as in the frequentist calculation.

Under an expected posterior probability approach, we again assume Beta(66,302) design priors to represent prior uncertainty about event probabilities. A sample size of 110 patients per group is required to provide 90% expected probability of non-inferiority. Because this approach controls the probability of concluding non-inferiority rather than the probability of achieving a significant result when testing for non-inferiority, it leads us to a much smaller sample size than under the Bayesian power approach (although assuming the same flat analysis priors) and also lower than under the frequentist approach.

**Figure 1:**
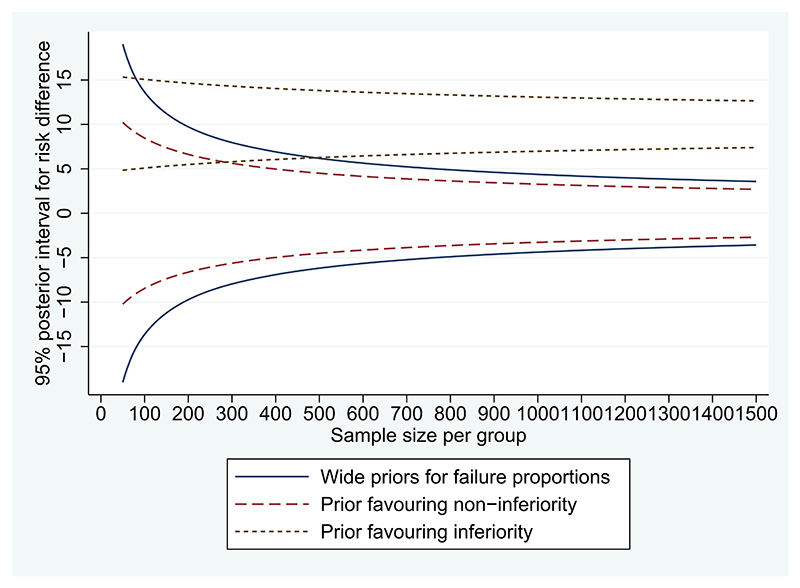
95% credible intervals for the risk difference as a function of sample size for an observed risk difference of 0%, assuming (as analysis priors) flat Beta (1,1) priors for failure proportions, a prior favoring non-inferiority of the experimental arm (Beta(11,48) priors for failure proportions) or a prior favoring inferiority (Beta(66,302) prior for control arm failure proportion, Beta(141,362) prior for experimental arm failure proportion)

Under a precision-based approach to determining sample size, we examine the relationship between 95% credible interval width and sample size, assuming an observed risk difference of 0% (Figure 1). Previous authors have proposed choosing sample size according to a desired credible interval width, or by discussing the trade-off between increased sampling costs and increased precision.^12^ For example, suppose that 12% is judged to be the maximum acceptable width for a 95% credible interval to be obtained for the risk difference comparing dolutegravir to standard of care. A sample size of 530 per group would provide this level of precision. Trial investigators could discuss whether it would be worth recruiting an extra 250 patients per group in order to reduce the anticipated interval width to 10%, for example, or an extra 650 patients per group to reduce the width to 8%, while taking into account the costs of including additional patients.

**Figure 2:**
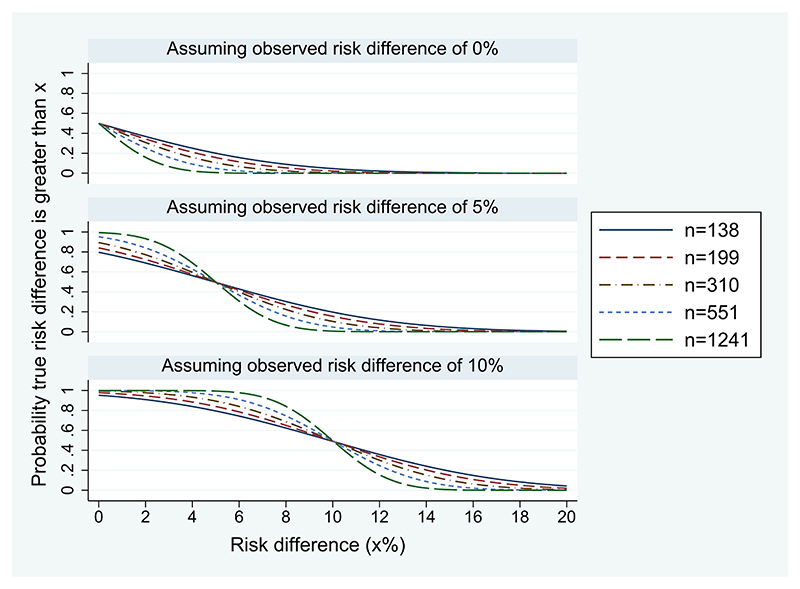
ACCEPT curves corresponding to various sample sizes *n* (per group): cumulative probability of the true risk difference being greater than a threshold value *x*%

Figure 2 presents ACCEPT curves corresponding to different plausible sample sizes and data. The sample sizes chosen are those providing 90% frequentist power for non-inferiority margins of 15% (138 per group), 12.5% (199 per group), 10% (310 per group), 7.5% (551 per group), and 5% (1241 per group), assuming 18% failure proportions in both groups. To plot an ACCEPT curve without observed trial data, we need to assume a hypothetical value for the observed risk difference; Figure 2 displays plots corresponding to 0%, 5%, and 10%.

In Figure 2, we see that the ACCEPT curves become steeper as the sample size increases. The steepest slope of each curve reflects the precision provided by the corresponding sample size and we can use these plots to compare posterior probability statements that could be made from trials of varying sample sizes. For example, if a risk difference of 0% was observed in a trial using the largest sample size shown (1241 patients per group), we would conclude there to be very low probability (<0.1) that the true risk difference is greater than 2%. Conversely, a trial using the smallest sample size shown (138 per group) would show very low probability (<0.1) that the true risk difference is greater than 6%. Although the curves become steeper as sample size increases, the change in shape is gradual and there is no clear distinction between curves which are more informative or easily interpreted and curves which provide little information. We therefore recommend considering these plots alongside the plot shown in Figure 1 rather than as a standalone tool for determining sample size.

### 3.1 Incorporating informative priors on the treatment effect

We now examine the potential impact of incorporating informative analysis prior distributions for the treatment effect on the predictive power of the trial. Suppose that an informative prior will be used in the planned Bayesian analysis, based on external evidence or expert opinion. As an extreme example, we choose Beta(11,48) analysis prior distributions for the failure proportions *π*_1_ and *π*_0_, with means of 18% and standard deviations of 5%, representing very strong prior opinion for non-inferiority (with prior probability 92%). We again consider the predictive power of a trial including 310 patients per group (based on the frequentist sample size calculation), and find that predictive power is now calculated as 90%. The additional information contributed by use of an informative prior favoring non-inferiority would mean that a smaller sample size is required to achieve an acceptable level of power in the Bayesian analysis.

When incorporating the analysis prior favoring non-inferiority in an expected posterior probability approach, the prior probability for non-inferiority is 92% so no patients are needed to provide an expected probability of more than 90%. Under a precision-based approach, using a sample size of 400 per group would provide a 95% interval width of 10% (Figure 1).

We now consider the impact of using a skeptical informative prior distribution in the analysis, representing strong prior opinion for inferiority of the experimental arm. We choose a Beta(66,302) analysis prior distribution for the control arm failure proportion *π*_0_, with mean 18% and SD 2%, and a Beta(141,362) distribution for the experimental arm failure proportion *π*_1_, with mean 28% and SD 2%. For a trial including 310 patients per group, the predictive power for a Bayesian analysis incorporating this skeptical analysis prior is calculated as only 41%. A sample of 760 per group would be required to provide 90% predictive power. When using an expected posterior probability approach and incorporating the skeptical prior, we find that a sample of 280 patients per group would be required to provide 90% expected probability of non-inferiority. Under a precision-based approach, the information represented by prior information alone corresponds to a 95% interval width of 11%. A sample size of 90 patients per group would provide a 95% interval width of 10%, while 350 patients per group provides a 95% interval width of 8% (Figure 1).

### 3.2 Incorporating an informative prior on the non-inferiority margin

Rather than assuming a fixed non-inferiority margin, we could place a prior on the margin to represent clinical uncertainty. In practice, a prior for the non-inferiority margin should ideally be based on formal elicitation of clinical opinion, representing variability of opinion among experts.^13^ Here, we use two example prior distributions for the purpose of demonstrating the impact of uncertainty around the non-inferiority margin on the predictive power of the ODYSSEY trial design. Initially we assume a Uniform(8%, 12%) prior and calculate the predictive power of a trial including 310 patients per group (based on the frequentist sample size calculation) as 82%, which is almost unchanged from the predictive power calculated assuming a fixed non-inferiority margin. With a wider Uniform(5%, 15%) prior, the predictive power for a trial of the same size is 78%. The predictive power allowing for uncertainty in the proportions is fairly linear with respect to the non-inferiority margin, so taking an average across varying non-inferiority margins makes little difference in this example design.

Similarly, placing a prior on the non-inferiority margin made no practical difference when calculating the expected posterior probability of non-inferiority. A sample size of 110 patients per group provides 90% expected probability of non-inferiority when assuming a Uniform(8%, 12%) prior for the non-inferiority margin, while 130 patients per group provides 90% expected probability under a Uniform(5%, 15%) prior.

## 4 Discussion

Bayesian analysis of a non-inferiority trial is advantageous in allowing direct probability statements to be made about the difference between treatments, rather than relying on an arbitrary and often poorly justified non-inferiority margin, and allowing inclusion of prior information on the treatment effect. When the primary analysis of a trial will be Bayesian, for consistency the sample size should ideally be determined using a Bayesian approach. Bayesian methods for sample size determination are less familiar to applied statisticians than frequentist methods and more rarely used in practice. In this article, we have discussed several approaches to Bayesian sample size determination for a non-inferiority trial and demonstrated their application to an example trial design. We now compare the advantages and disadvantages of these approaches.

A Bayesian power approach is appealing as it is analogous to the standard frequentist approach to sample size determination and is straightforward to implement and communicate. This approach will always lead to larger sample sizes than an equivalent frequentist approach unless an informative prior is specified for the treatment effect, because allowance is made for the uncertainty in the design parameters. In the ODYSSEY example, allowing for a 2% SD in the assumed failure proportions would require sample size to be increased slightly (by 13%) from that giving 90% frequentist power to achieve 80% predictive power or increased substantially (by 90%) to achieve 90% predictive power. The increased sample size is required to avoid making an unrealistic assumption that the failure proportions are fixed and known, which can potentially result in an underpowered trial. The increase proposed in this example is similar to sample size increases often made to provide resilience in a trial design based on fixed parameters. Uncertainty over the magnitude of both treatment effect and control group risk is taken into account, where lower treatment effect or higher control group risk leads to lower power. An alternative approach to addressing the design issues arising from uncertainty in the control group risk is to define a “non-inferiority frontier” (where the margin varies with control risk) rather than a fixed non-inferiority margin.^21^

Under an expected posterior probability approach, sample size is determined by examining the probability of non-inferiority occurring in the trial. This approach leads to smaller sample sizes than a Bayesian power approach, but would be appropriate only when the focus of the trial is on estimating the posterior probability of non-inferiority rather than on testing. This can be justified in preference to a conventional hypothesis testing trial when evaluating treatments for uncommon conditions, for example, or when a decision has to be made on the basis of a planned trial and the achievable sample size is limited.^22^ Choosing an expected posterior probability approach expressly to obtain a lower required sample size would however not be recommended. A precision-based approach based on confidence interval width could also be useful in settings where the sample size of a planned trial is restricted by limits on recruitment or costs, but it would be difficult to base sample size decisions on this approach alone. In frequentist sample size calculations, precision-based approaches are sometimes used to express how much precision is available for estimating a parameter of secondary interest, but rarely used in the primary calculation.

Throughout the article, we have assumed conjugate Beta distributions to represent prior information about failure proportions in the trial arms. When assessing the relationship between sample size and credible interval width, this choice allows analytical calculation of credible intervals. If instead we placed priors on the treatment effect and control arm proportions, the precision-based approach would be more complicated to implement, typically requiring simulations, but the Bayesian power approach and expected posterior probability approach could still be calculated as outlined above. The precision-based approach could also be extended to incorporate a design prior and would then need to be implemented using simulations. For simplicity, we have used a normal approximation for the posterior distribution of the treatment effect. We have assumed equal allocation between trial arms throughout, but the formulae could be adapted for unequal allocation. The analysis priors favoring non-inferiority or inferiority were both chosen as extreme examples, to illustrate the potential influence of an informative prior on sample size calculations, rather than as realistic choices in practice. However, in settings where recruitment is difficult, such as rare diseases or pediatric trials, it may be considered appropriate to borrow information from a relevant external trial.^6^ An informative analysis prior could then be used to represent information from external data and the planned sample size would be reduced accordingly.

Determining sample size by considering the predictive probability of obtaining a significant result was proposed first by Spiegelhalter and Freedman^7^ and subsequently developed further.^8–10^ Here, we have assumed that a Bayesian analysis is planned, but this approach can also be used when the primary analysis will be frequentist and is then referred to as a hybrid Bayesian-frequentist approach. We focused on the joint probability that the trial will produce a significant Bayesian result and that this decision is correct, rather than on the unconditional probability of a significant Bayesian result averaged over the entire parameter range. Since the prior we used at the design stage was supportive of non-inferiority, the difference between the two probabilities was negligible. If a prior giving substantial support to inferiority were used, the joint probability would be reduced and we would suggest comparing the Bayesian power against a lower threshold than the usual 90%. Alternatively, the Bayesian power approach could be defined using the power conditional on non-inferiority being correct.^8^ Joseph et al proposed choosing sample size on the basis of precision rather than power, by considering interval width for a fixed coverage probability, level of coverage provided for a fixed interval width, or a combination of interval width and coverage.^11^ Rather than focusing on precision in estimation, Lindley recommended taking a decision-theoretic approach to choosing sample size, by specifying a utility function to represent the benefit and cost of running a study of a certain size, and then choosing sample size to maximize the expected utility.^12^

A decision-theoretic approach to choosing sample size in non-inferiority trials was proposed by Bouman et al and compared with a frequentist approach.^23^ A health economic decision model was constructed to calculate the expected value of sampling information and expected net benefit of sampling for different possible sample sizes, and their methods were applied to design of a trial comparing individualized vs standard duration of elastic compression stocking therapy for prevention of post-thrombotic syndrome. Bouman et al argue strongly in favor of a decision-theoretic approach over a frequentist approach on the grounds that uncertainty is fully acknowledged, costs and adverse effects of treatment are taken into account as well as the main therapeutic benefit, and the value of the planned trial to society is considered. A disadvantage of taking a decision-theoretic approach is that developing an appropriate decision model and collecting information to provide inputs to the model is likely to require a considerable amount of time and resources, and would not usually be feasible in practice unless separate funding is obtained for the design stage of the trial.

An alternative Bayesian view is that an advance sample size calculation is not necessarily required; in an open trial, recruitment continues until investigators agree that a conclusion can be drawn from the accumulated evidence.^24,25^ This approach involves performing multiple analyses of the data, which is not problematic in a fully Bayesian framework.^26^ This approach has been more commonly used in exploratory and early-phase trials than in confirmatory trials.^27^ However, it was used recently in the high-profile confirmatory REMAP-CAP trial evaluating interventions for treating community-acquired pneumonia, which has been adapted to include evaluation of interventions suitable for treating patients with COVID-19.^28^ Obtaining funding for a trial without a pre-planned sample size may be difficult, unless the trial is carried out as part of an ongoing program of research.^24^

Bayesian methods offer several advantages for analysis of non-inferiority trials, and we have demonstrated and compared corresponding Bayesian approaches to sample size determination. A Bayesian power approach to sample size determination would be most accessible in practical settings, because it is analogous to the standard frequentist approach and straightforward to implement and communicate. Sample sizes will be larger than under frequentist calculations unless an informative prior is specified for the treatment effect, because the Bayesian approach makes appropriate allowance for uncertainty in the assumed input parameters. Arguably this provides resilience against unanticipated variation in unknown event rates, which can be particularly problematic in non-inferiority trials where the frequentist paradigm leads to a binary non-inferiority conclusion on the basis of the limit of the 95% confidence interval. An expected posterior probability approach can lead to smaller sample sizes and is appropriate in settings where the focus is on estimating the posterior probability of non-inferiority rather than on testing.

## Supplementary Material

Supplementary material

## Figures and Tables

**Table 1:** Summary of characteristics of the sample size determination approaches

	Approach to sample size determination			
	Bayesian power	Expected posterior probability	Precision-based	Frequentist
Judgment on which sample size choice based	Predictive power required	Mean posterior probability of NI required	Precision required	Power required
Key output from planned analysis	Whether NI is highly likely	Posterior probability of NI	Precision of treatment effect	Whether confidence is high for NI
Allows for uncertainty in design parameters?	Yes	Yes	No	No
Allows for inclusion of prior knowledge on treatment effect?	Yes	Yes	Yes	No
Typical value for sample size	Higher than frequentist	Lower than frequentist	Depends on precision	-
Example of suitable use	Confirmatory trials; regulatory submission	Uncommon diseases; exploratory trials	Treatment effect fed into further modeling	Confirmatory trials; regulatory submission

Abbreviation: NI, non-inferiority.

**Table 2:** Results obtained from different sample size determination approaches for the ODYSSEY trial example

Prior to be used in analysis	Approach to sample size determination			
	Bayesian power	Expected posterior probability	Precision-based	Frequentist
N/A	-	-	-	310 per group provides 90% power
Wide: Beta(11,48) priors for failure proportions	440 per group provides 90% power	110 per group provides 90% probability	450 per group provides 95% interval width of 10%	-
Enthusiastic (favors non-inferiority)	310 per group provides 90% power	0 per group provides 92% probability	400 per group provides 95% interval width of 10%	-
Skeptical (favors inferiority)	760 per group provides 90% power	280 per group provides 90% probability	90 per group provides 95% interval width of 10%	-

## Data Availability

Data sharing is not applicable to this article as no data sets were created or analysed in this study.

## References

[1] Rehal S, Morris TP, Fielding K, Carpenter JR, Phillips PP (2016). Non-inferiority trials: are they inferior? A systematic review of reporting in major medical journals. BMJ Open.

[2] Ghosh P, Nathoo F, Gonen M, Tiwari RC (2011). Assessing noninferiority in a three-arm trial using the Bayesian approach. Stat Med.

[3] Gamalo MA, Tiwari RC, LaVange LM (2014). Bayesian approach to the design and analysis of non-inferiority trials for anti-infective products. Pharm Stat.

[4] Gamalo-Siebers M, Gao A, Lakshminarayanan M (2016). Bayesian methods for the design and analysis of noninferiority trials. J Biopharm Stat.

[5] Ghosh S, Ghosh S, Tiwari RC (2016). Bayesian approach for assessing non-inferiority in a three-arm trial with pre-specified margin. Stat Med.

[6] Hampson LV, Whitehead J, Eleftheriou D, Brogan P (2014). Bayesian methods for the design and interpretation of clinical trials in very rare diseases. Stat Med.

[7] Spiegelhalter DJ, Freedman LS (1986). A predictive approach to selecting the size of a clinical-trial, based on subjective clinical opinion. Stat Med.

[8] Spiegelhalter DJ, Abrams KR, Myles JP (2004). Bayesian Approaches to Clinical Trials and Health-Care Evaluation.

[9] O’Hagan A, Stevens JW (2001). Bayesian assessment of sample size for clinical trials of cost-effectiveness. Med Decis Making.

[10] O’Hagan A, Stevens JW, Campbell MJ (2005). Assurance in clinical trial design. Pharm Stat.

[11] Joseph L, du Berger R, Belisle P (1997). Bayesian and mixed Bayesian/likelihood criteria for sample size determination. Stat Med.

[12] Lindley DV (1997). The choice of sample size. J R Stat Soc D.

[13] Aupiais C, Alberti C, Schmitz T, Baud O, Ursino M, Zohar S (2019). A Bayesian non-inferiority approach using experts’ margin elicitation application to the monitoring of safety events. BMC Med Res Methodol.

[14] Kunzmann K, Grayling MJ, Lee KM, Robertson DS, Rufibach K, Wason JMS (2021). A review of Bayesian perspectives on sample size derivation for confirmatory trials. Am Stat.

[15] Wang F, Gelfand AE (2002). A simulation-based approach to Bayesian sample size determination for performance under a given model and for separating models. Stat Sci.

[16] Brutti P, De Santis F, Gubbiotti S (2008). Robust Bayesian sample size determination in clinical trials. Stat Med.

[17] Clements MN, White IR, Copas AJ (2022). Improving clinical trial interpretation with ACCEPT analyses. NEJM Evid.

[18] Moore CL, Turkova A, Mujuru H (2021). ODYSSEY clinical trial design: a randomised global study to evaluate the efficacy and safety of dolutegravir-based antiretroviral therapy in HIV-positive children, with nested pharmacokinetic sub-studies to evaluate pragmatic WHO-weight-band based dolutegravir dosing. BMC Infect Dis.

[19] Amuge P, Lugemwa A, Wynne B (2022). Once-daily dolutegravir-based antiretroviral therapy in infants and children living with HIV from age 4 weeks: results from the below 14kg cohort in the randomised ODYSSEY trial. Lancet HIV.

[20] Morita S, Thall PF, Muller P (2008). Determining the effective sample size of a parametric prior. Biometrics.

[21] Quartagno M, Walker AS, Babiker AG (2020). Handling an uncertain control group event risk in non-inferiority trials: non-inferiority frontiers and the power-stabilising transformation. Trials.

[22] Cornelius V, Elkes J, White IR A Bayesian framework for non-inferioirty evaluation in randomised controlled trials of uncommon conditions.

[23] Bouman AC, ten Cate-Hoek AJ, Ramaekers BLT, Joore MA (2015). Sample size estimation for non-inferiority trials: frequentist approach versus decision theory approach. PLoS One.

[24] Lilford RJ, Thornton JG, Braunholtz D (1995). Clinical trials and rare diseases: a way out of a conundrum. BMJ.

[25] Harrell F Continuous learning from data: no multiplicities from computing and using Bayesian posterior probabilities as often as desired.

[26] Spiegelhalter DJ, Freedman LS, Parmar MKB (1994). Bayesian approaches to randomized trials. J R Stat Soc A.

[27] Ryan EG, Brock K, Gates S, Slade D (2020). Do we need to adjust for interim analyses in a Bayesian adaptive trial design?. BMC Med Res Methodol.

[28] Angus DC, Berry S, Lewis RJ (2020). The REMAP-CAP (randomized embedded multifactorial adaptive platform for community-acquired pneumonia) study rationale and design. Ann Am Thorac Soc.

