# Supplementary material for: Practical approaches to Bayesian sample size determination in non-inferiority trials with binary outcomes
Source: Stat Med. Author manuscript; Available in PMC 2024 Mar 12. (PMC7615731; doi:10.1002/sim.9661)
Supplement: Supplementary material [file EMS194527-supplement-Supplementary_material.pdf]

Practical approaches to Bayesian sample size determination in non-inferiority trials with binary outcomes: Supplementary material

Implementing Bayesian power approach in ODYSSEY trial application: WinBUGS code

```
model  {

# NI margin delta
delta <- 0.10
# Sample size per arm
n <- 310

# Sampling from priors assumed for failure proportions at design stage
alphaD <- 66
betaD <- 302
pi1 ~ dbeta(alphaD,betaD)
pi2 ~ dbeta(alphaD,betaD)
r1 ~ dbin(pi1,n)
r2 ~ dbin(pi2,n)
p1 <- r1/n
p2 <- r2/n
diff <- p1-p2
var <- (p1*(1-p1)/n) + p2*(1-p2)/n

# Incorporating wide analysis priors to obtain posterior distributions for failure
proportions
alphaA1 <- 1
betaA1 <- 1
pi1_post1mean <- (alphaA1+r1)/(alphaA1+betaA1+n)
pi2_post1mean <- (alphaA1+r2)/(alphaA1+betaA1+n)
pi1_post1var <- (pi1_post1mean*(1-pi1_post1mean)) / (alphaA1+betaA1+n-1)
pi2_post1var <- (pi2_post1mean*(1-pi2_post1mean)) / (alphaA1+betaA1+n-1)
diff_post1 <-  pi1_post1mean - pi2_post1mean
var_post1 <- pi1_post1var + pi2_post1var

# Rejection of null hypothesis in Bayesian analysis using wide priors
rejection_wideprior <- step(-1.96 + (delta - diff_post1)/sqrt(var_post1) )*(1-
step(pi1-pi2-delta))

# Rejection of null hypothesis in Bayesian analysis using wide priors,
incorporating uncertainty about NI margin
deltav ~ dunif(0.08,0.12)
rejection_wideprior_deltav <- step(-1.96 + (deltav - diff_post1)/sqrt(var_post1)
)*(1-step(pi1-pi2-delta))

# Incorporating analysis priors favouring non-inferiority to obtain posterior
distributions for failure proportions
alphaA2 <- 11
betaA2 <- 48
pi1_post2mean <- (alphaA2+r1)/(alphaA2+betaA2+n)
pi2_post2mean <- (alphaA2+r2)/(alphaA2+betaA2+n)
pi1_post2var <- (pi1_post2mean*(1-pi1_post2mean)) / (alphaA2+betaA2+n-1)
pi2_post2var <- (pi2_post2mean*(1-pi2_post2mean)) / (alphaA2+betaA2+n-1)
diff_post2 <-  pi1_post2mean - pi2_post2mean
var_post2 <- pi1_post2var + pi2_post2var

# Rejection of null hypothesis in Bayesian analysis using priors favouring non-
inferiority
rejection_favNIprior <- step(-1.96 + (delta - diff_post2)/sqrt(var_post2) )*(1-
step(pi1-pi2-delta))

# Incorporating analysis priors favouring inferiority to obtain posterior
distributions for failure proportions
alphaA3 <- 141
betaA3 <- 362
```

```
alphaA4 <- 66
betaA4 <- 302
pi1_post3mean <- (alphaA3+r1)/(alphaA3+betaA3+n)
pi2_post3mean <- (alphaA4+r2)/(alphaA4+betaA4+n)
pi1_post3var <- (pi1_post3mean*(1-pi1_post3mean)) / (alphaA3+betaA3+n-1)
pi2_post3var <- (pi2_post3mean*(1-pi2_post3mean)) / (alphaA4+betaA4+n-1)
diff_post3 <-  pi1_post3mean - pi2_post3mean
var_post3 <- pi1_post3var + pi2_post3var

# Rejection of null hypothesis in Bayesian analysis using priors favouring
inferiority
rejection_favIprior <- step(-1.96 + (delta - diff_post3)/sqrt(var_post3) )*(1-
step(pi1-pi2-delta))

# Outputs to monitor
m[1] <- rejection_wideprior
m[2] <- rejection_wideprior_deltav
m[3] <- rejection_favNIprior
m[4] <- rejection_favIprior

}
```

Implementing expected posterior probability approach in ODYSSEY trial application: WinBUGS code

```
model  {

# NI margin delta
delta <- 0.10
# Sample size per arm
n <-310

# Sampling from priors assumed for failure proportions at design stage
alphaD <- 66
betaD <- 302
pi1 ~ dbeta(alphaD,betaD)
pi2 ~ dbeta(alphaD,betaD)
r1 ~ dbin(pi1,n)
r2 ~ dbin(pi2,n)
p1 <- r1/n
p2 <- r2/n
diff <- p1-p2
var <- (p1*(1-p1)/n) + p2*(1-p2)/n

# Incorporating wide analysis priors to obtain posterior distributions for failure
proportions
alphaA1 <- 1
betaA1 <- 1
pi1_post1mean <- (alphaA1+r1)/(alphaA1+betaA1+n)
pi2_post1mean <- (alphaA1+r2)/(alphaA1+betaA1+n)
pi1_post1var <- (pi1_post1mean*(1-pi1_post1mean)) / (alphaA1+betaA1+n-1)
pi2_post1var <- (pi2_post1mean*(1-pi2_post1mean)) / (alphaA1+betaA1+n-1)
diff_post1 <-  pi1_post1mean - pi2_post1mean
var_post1 <- pi1_post1var + pi2_post1var

# Posterior probability in Bayesian analysis using wide priors
postprob_wideprior <- phi(  (delta-diff_post1)/sqrt(var_post1) )

# Posterior probability in Bayesian analysis using wide priors, incorporating
uncertainty about NI margin
deltav ~ dunif(0.08,0.12)
postprob_wideprior_deltav <- phi(  (deltav-diff_post1)/sqrt(var_post1) )

# Incorporating analysis priors favouring non-inferiority to obatin posterior
distributions for failure proportions
```

```
alphaA2 <- 11
betaA2 <- 48
pi1_post2mean <- (alphaA2+r1)/(alphaA2+betaA2+n)
pi2_post2mean <- (alphaA2+r2)/(alphaA2+betaA2+n)
pi1_post2var <- (pi1_post2mean*(1-pi1_post2mean)) / (alphaA2+betaA2+n-1)
pi2_post2var <- (pi2_post2mean*(1-pi2_post2mean)) / (alphaA2+betaA2+n-1)
diff_post2 <-  pi1_post2mean - pi2_post2mean
var_post2 <- pi1_post2var + pi2_post2var

# Posterior probability in Bayesian analysis using priors favouring non-inferiority
postprob_favNIprior <- phi(  (delta-diff_post2)/sqrt(var_post2) )

# Incorporating analysis priors favouring inferiority to obtain posterior
distributions for failure proportions
alphaA3 <- 141
betaA3 <- 362
alphaA4 <- 66
betaA4 <- 302
pi1_post3mean <- (alphaA3+r1)/(alphaA3+betaA3+n)
pi2_post3mean <- (alphaA4+r2)/(alphaA4+betaA4+n)
pi1_post3var <- (pi1_post3mean*(1-pi1_post3mean)) / (alphaA3+betaA3+n-1)
pi2_post3var <- (pi2_post3mean*(1-pi2_post3mean)) / (alphaA4+betaA4+n-1)
diff_post3 <-  pi1_post3mean - pi2_post3mean
var_post3 <- pi1_post3var + pi2_post3var

# Posterior probability in Bayesian analysis using priors favouring inferiority
postprob_favIprior <-  phi(  (delta-diff_post3)/sqrt(var_post3) )

# Outputs to monitor
m[1] <- postprob_wideprior
m[2] <- postprob_wideprior_deltav
m[3] <- postprob_favNIprior
m[4] <- postprob_favIprior

}
```

Implementing precision-based approach in ODYSSEY trial application: Stata code

Plot posterior intervals for the risk difference as a function of sample size

```
* Parameters defining posterior distribution when assuming flat priors: Beta(1,1)
distributions for each failure proportion
scalar a1=1
scalar b1=1
scalar p1=0.5
* Parameters defining posterior distribution when assuming an informative prior
favouring non-inferiority: Beta(11,48) distributions for each failure proportion
scalar a2=11
scalar b2=48
scalar p2=0.18
* Parameters defining posterior distribution when assuming an informative prior
favouring inferiority: Beta(66,302) distribution for control arm, Beta(141,362)
distribution for intervention arm
scalar a3=66
scalar b3=302
scalar a4=141
scalar b4=362
scalar p3=0.18
scalar p4=0.28
* Combined plot of 95% credible intervals for the risk difference as a function of
sample size for an observed risk difference of 0%, assuming each of three priors
twoway function y1=100*(1.96*sqrt((2*a1*(b1+x)+(x+b1-a1)*(p1*x+p1*x)-
((p1*x)^2+(p1*x)^2))/(((a1+b1+x)^2)*(a1+b1+x+1)))), range(50 1500) xlab(0(100)1500)
ylab(-15(5)15) lcolor(navy) || function y2=100*(-1.96*sqrt((2*a1*(b1+x)+(x+b1-
a1)*(p1*x+p1*x)-((p1*x)^2+(p1*x)^2))/(((a1+b1+x)^2)*(a1+b1+x+1)))), range(50 1500)
lcolor(navy) || function y1=100*(1.96*sqrt((2*a2*(b2+x)+(x+b2-a2)*(p2*x+p2*x)-
```

```
((p2*x)^2+(p2*x)^2))/(((a2+b2+x)^2)*(a2+b2+x+1)))), range(50 1500) lcolor(maroon)
lpattern(dash) || function y2=100*(-1.96*sqrt((2*a2*(b2+x)+(x+b2-a2)*(p2*x+p2*x)-
((p2*x)^2+(p2*x)^2))/(((a2+b2+x)^2)*(a2+b2+x+1)))), range(50 1500) lcolor(maroon)
lpattern(dash) ||  function y1=100*( ((a4+x*p4)/(a4+b4+x)) - ((a3+x*p3)/(a3+b3+x))
+ 1.96*sqrt( ((a3+x*p3)*(b3+x-x*p3))/(((a3+b3+x)^2)*(a3+b3+x+1)) +
((a4+x*p4)*(b4+x-x*p4))/(((a4+b4+x)^2)*(a4+b4+x+1)) ) ), range(50 1500)
lcolor(olive) lpattern(shortdash) ||  function y2=100*( ((a4+x*p4)/(a4+b4+x)) -
((a3+x*p3)/(a3+b3+x)) - 1.96*sqrt( ((a3+x*p3)*(b3+x-
x*p3))/(((a3+b3+x)^2)*(a3+b3+x+1)) + ((a4+x*p4)*(b4+x-
x*p4))/(((a4+b4+x)^2)*(a4+b4+x+1)) ) ), range(50 1500) lcolor(olive)
lpattern(shortdash) legend(order(1 3 5) col(1) label(1 "Wide priors for failure
proportions") label(3 "Prior favouring non-inferiority") label(5 "Prior favouring
inferiority")) xtitle("Sample size per group") ytitle("95% posterior interval for
risk difference")
```

Plot non-inferiority acceptability curves for a range of sample sizes

```
* Calculate sample sizes providing 90% power under frequentist sample size
calculations for five different NI margins between 5% and 15%

set obs 5
input margin
0.15
0.125
0.10
0.075
0.05
gen n=round(((invnorm(0.975)+invnorm(0.9))^2)*(0.18*(1-0.18)+0.18*(1-0.18))/((0
.18-0.18-margin)^2))


* Calculate posterior means and SDs for an observed risk difference of zero,
assuming flat priors: Beta(1,1) distributions for each failure proportion

scalar a=1
scalar b=1
scalar p0=0.5
scalar p1=0.5
gen mean=(a+p1*n)/(a+b+n)-(a+p0*n)/(a+b+n)
gen sd=sqrt((2*a*(b+n)+(n+b-a)*(p0*n+p1*n)-
((p0*n)^2+(p1*n)^2))/(((a+b+n)^2)*(a+b+n+1)))

forvalues i=1/5 {
	scalar mean`i'= 100*mean in `i'
	scalar sd`i' = 100*sd in `i'
}


* Plot non-inferiority acceptability curves for varying sample size, for an
observed risk difference of zero

twoway function y1=(1-normal((x-mean1)/sd1)), range(0 20) xlab(0(2)20)
ylab(0(0.2)1) || function y2=(1-normal((x-mean2)/sd2)), range(0 20) || function
y3=(1-normal((x-mean3)/sd3)), range(0 20) || function y4=(1-normal((x-mean4)/sd4)),
range(0 20) || function y5=(1-normal((x-mean5)/sd5)), range(0 20) legend(label(1
"n=138") label(2 "n=199") label(3 "n=310") label(4 "n=551") label(5 "n=1241"))
xtitle("Risk difference (x%)") ytitle("Probability true risk difference is greater
than x") title("Assuming observed risk difference of 0%")
```
